# Climate-driven decline of Norway spruce in Central Europe: a threshold crossing in the warming Carpathian Basin

**DOI:** 10.1007/s00484-026-03174-9

**Published:** 2026-04-14

**Authors:** Zsuzsa Lisztes-Szabó, Albert Tóth, Anna F. Filep, Olivér Szentes, Elemér László, Mihály Braun

**Affiliations:** 1 HUN-REN Institute for Nuclear Research, Bem tér 18/C, Debrecen, 4026 Hungary; 2 Department of Botany, Faculty of Science and Technology, University of Debrecen, Egyetem tér 1., Debrecen, 4032 Hungary; 3 Pál Juhász-Nagy Doctoral School of Biology and Environmental Sciences, University of Debrecen, Egyetem tér 1., Debrecen, 4032 Hungary; 4 Climate Research Department, Hungaromet Hungarian Meteorological Service, Kitaibel Pál u.1, Budapest, 1024 Hungary

**Keywords:** Accelerated climate change, Citizen science, Continental basin, Extreme climate events, *Picea abies*, Tree decline

## Abstract

Norway spruce (*Picea abies* (L.) Karst.) is an ecologically and economically important conifer species that naturally dominates European boreal forests, the subalpine regions of the Alps, and the Carpathians. It is also widely planted beyond its native distribution area. In 2023, a significant spruce dieback was detected in the Carpathian Basin. To assess the extent of the damage, self-reported data were collected from citizens across Hungary about street, garden, and park spruce trees, covering all counties. A total of 4,081 spruce trees were reported in 2023, of which 2,422 (59.1%) died or showed signs of disease, particularly in areas where summer temperature anomalies exceeded 1 °C. We found a significant positive association (Spearman/Kendall correlation) between mortality rate and mean summer temperatures in previous years, and a negative association with the sum of annual precipitation. The increased spruce mortality in 2023 represents an intensification of a long-term trend that has also been affecting planted spruce forests for decades. The mean annual temperature in the Carpathian Basin seems to be approaching a threshold that exceeds the climatic tolerance of the species. Outside its natural range, the potential shift of areas suitable for planting this species may serve as an indicator of climate change, with ecological shifts progressing faster than previously anticipated. Environmental changes are already visible in the Carpathian Basin, underscoring that continental basins are at heightened risk of climate change. The Norway spruce dieback serves as an example, demonstrating the ecological and economic impacts of species loss due to climate change. Until a global solution is reached, local actions, supported by regional and precise climate models, will be crucial.

**Supplementary Information:**

The online version contains supplementary material available at 10.1007/s00484-026-03174-9.

## Main text

Due to global tree dieback, understanding the factors driving changes in temperate coniferous forest stands is increasingly crucial (Rivers et al. 2023). Gymnosperms were historically restricted to cold, nutrient-poor habitats by angiosperms (Bond 1989). As a result, conifer stands at the edges of their distribution area and in timberline zones are sensitive indicators of micro-scale environmental changes (Kupka et al. 2023; Popa et al. 2024; Zhang et al. 2006, 2022). Boreal conifer forests also play a significant role in carbon storage (Ivanov and Kurbatova 2023; Pan et al. 2024) and preserve rare microhabitats, acting as biodiversity refugia (Christiansen et al. 2022).

**Fig. 1:**
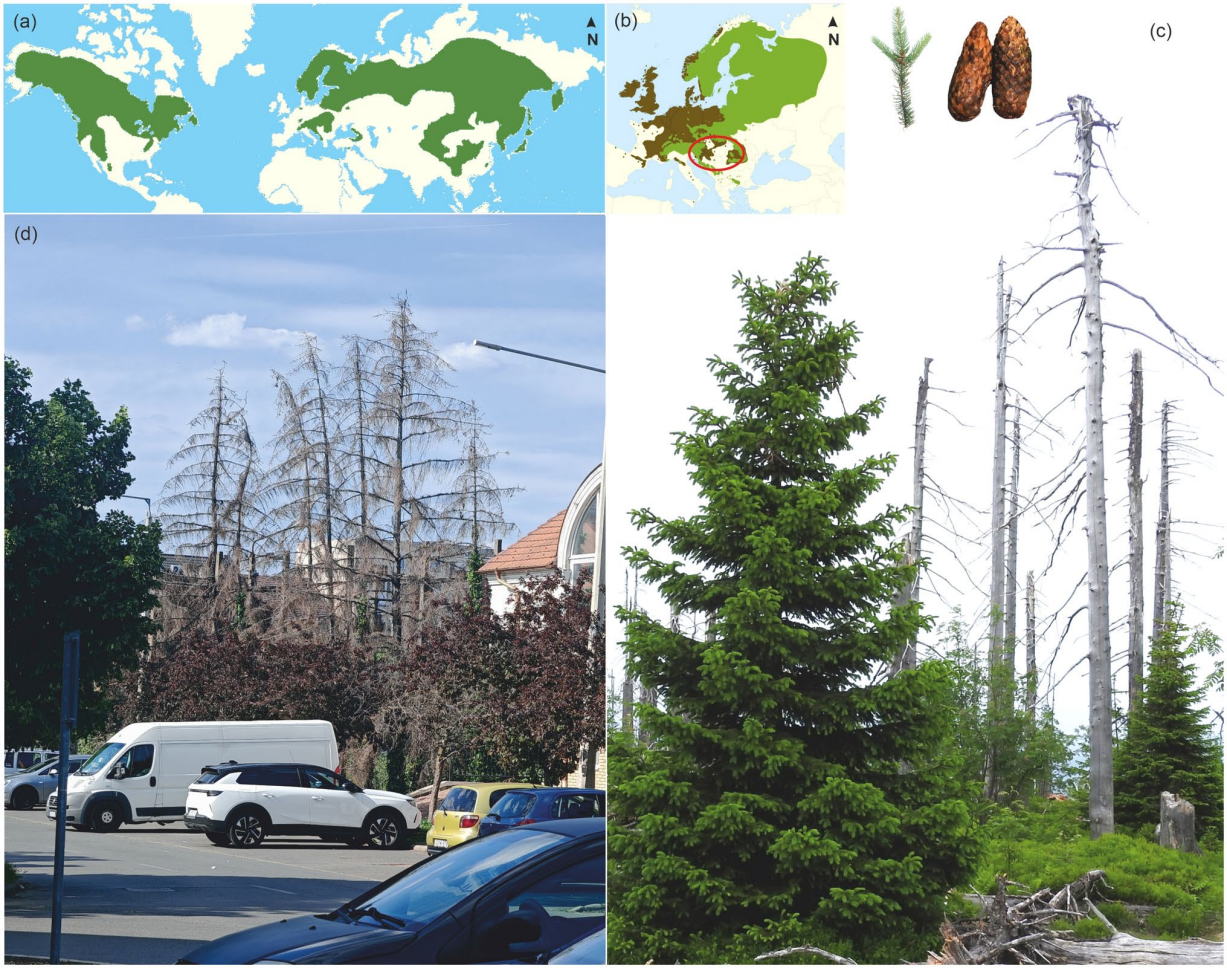
(**a**) Distribution of the spruce (*Picea*) genus and (**b**) Norway spruce (*Picea abies*) (Vidakovic 1991). The natural range of Norway spruce is shown in green; planted areas are marked in dark brown (OECD 2006). The red ellipse highlights the Carpathian Basin. The map is adapted from Caudullo et al. (2017). (**c**) Forest regeneration in the Šumava Mountains (Czech Republic) after a 2006 bark beetle outbreak (inset pictures: needles and cones). (**d**) Died spruce trees along a street in a town in the Carpathian Basin (Berettyóújfalu, Hungary)

The health of forest stands of the spruce (Picea) genus, which play a considerable economic and ecological role, is rapidly declining (Fig. 1; Supplementary Material S1). In recent years, the spread of diseases affecting spruce trees has increased worldwide (Hais and Kučera 2008; Huang et al. 2021; Lyon 2019; Kermavnar et al. 2023; Pócs 2022; Souza Lima et al. 2024). The most important species of the genus in Europe is Norway spruce (*Picea abies* (L.) Karst.), which expanded its range from southern European refugia during the Quaternary interglacials (Tjoelker et al. 2007). As its distribution history and ecological needs suggest, amphistomatic needles reflect its adaptation to cooler, continental climates (Srodon and Tobolski 2007). This study is looking for a potential association between the extreme climate conditions of two years (2022 and 2023) and the Norway spruce death in the Carpathian Basin (Fig. 1), highlighting its potential as an early warning indicator.

**Fig. 2:**
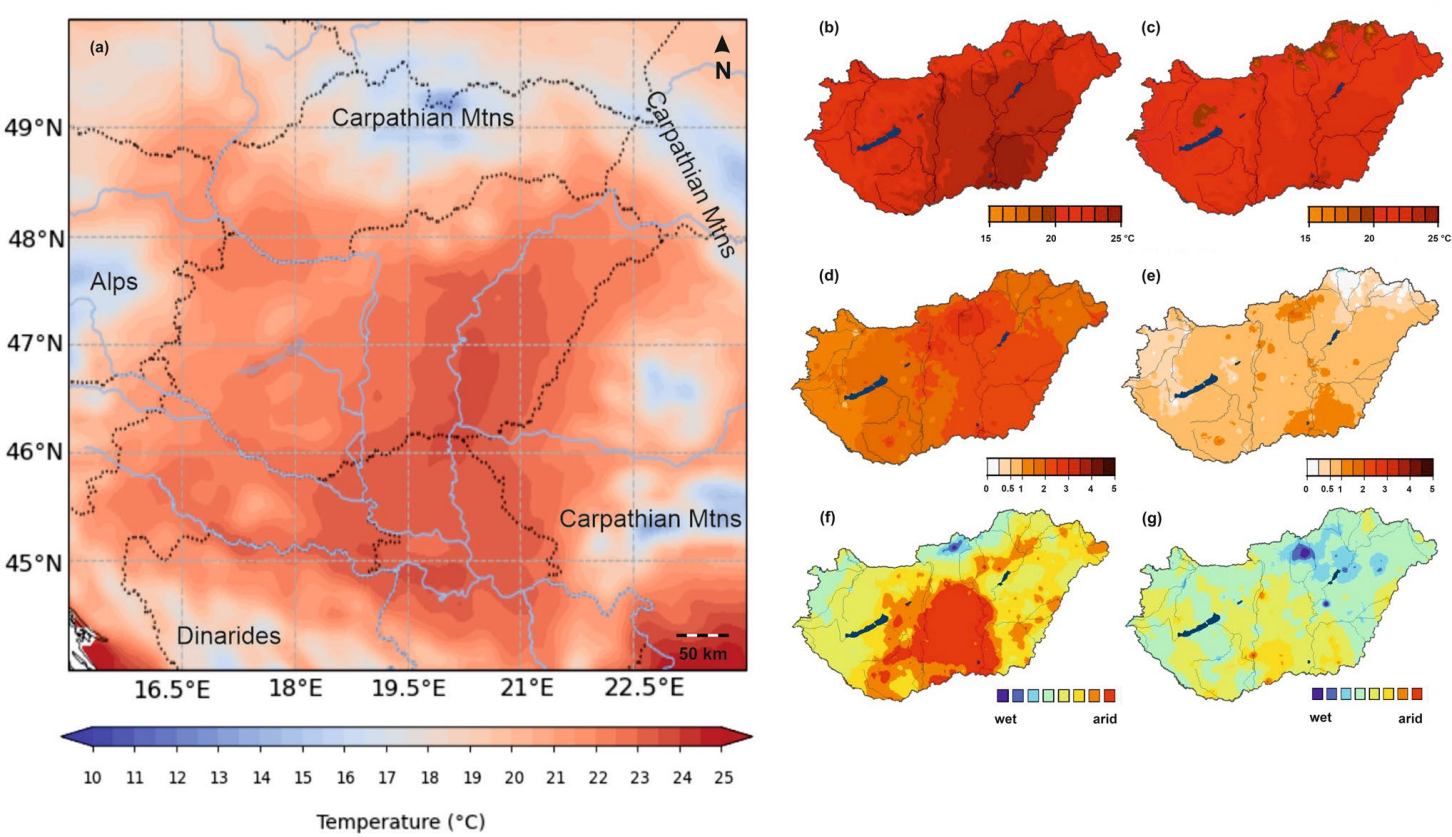
(**a**) The mean temperature (°C) of summer months in the Carpathian Basin in 2023. Graphically reproduced map based on ERA5-Land data (Muñoz-Sabater et al. 2021). (**b**) Mean summer temperature (°C) in Hungary in 2022 and (**c**) in 2023. (**d**) Temperature anomaly (°C) for the period 1991–2020 in 2022 and (**e**) 2023. (**f**) Standardized Precipitation Index in July 2022 and (**g**) June 2023 for interpretation of drought in summer months (calculated on World Meteorological Organization [WMO] 2012). The figures are reproduced based on the graphics of HungaroMet (2024)

In 2023, the rapid decline of spruce trees became evident in streets, parks, and gardens across the continental climate Carpathian Basin. We aimed to quantify the 2023 dieback and reveal potential association with weather severity. The warming climate of the Carpathian Basin is characterized by the fact that the average temperature in 2023 was 12.2 °C, 1.5 °C above the 1991–2020 norms. The summer was 0.8 °C warmer than the long-term average. The annual mean temperature has risen by + 1.5 °C since 1901, with changes ranging from + 1.2 °C to + 1.9 °C (HungaroMet 2024), at a 90% confidence level (Fig. 2).

In light of these climatic conditions, our aim was to quantify the 2023 dieback. To directly estimate the spruce dieback rate, we asked citizens contacted through their local municipalities to complete a Google questionnaire. The questions focused on how many spruce trees were present around the respondent’s household (in the garden or on the street), and how many had died or showed signs of decline during 2023. In addition, we collected the postal code of the respondent’s place of residence. The form was available for one month (January 2024). Details on the distribution of the questionnaire and additional methodological information are provided in Supplementary Material S2.

We excluded certain responses from the evaluation, such as those reporting more than 50 trees, because these were interpreted as representing forest monocultures rather than individual garden or street trees. We then calculated the association between spruce mortality (both the number and percentage of dead trees) and meteorological variables (temperature, precipitation, and anomalies) for the 1991–2020 period, as well as for 2022 and 2023. Significant Spearman’s (ρ) and Kendall’s (τ) rank correlations were assessed using temperature and precipitation data that were aggregated in several ways to enable comparisons across 19 counties (Past 2.17c, Hammer et al. 2001; see Supplementary Material S2 and S5 for details). In addition, statistical data on planted Norway spruce stands in Hungary were reviewed and discussed (see Supplementary Material S3 and S6).

**Fig. 3:**
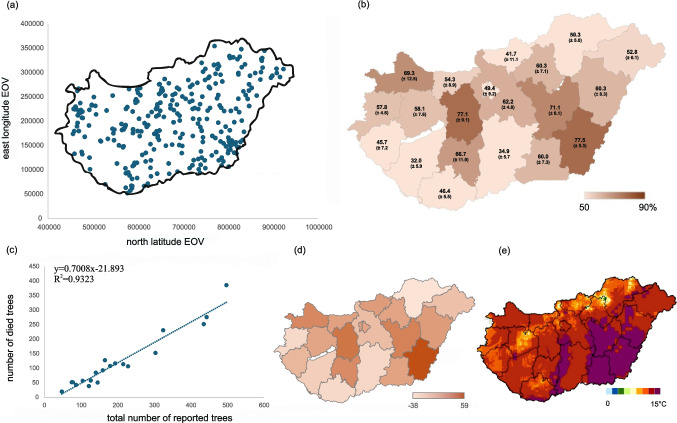
(**a**) EOV (Hungarian Unified National Projection coordinate system) positions representing the Hungarian settlements from which responses were received in January 2024. (**b**) Average spruce mortality rate per county. (**c**) The number of spruce trees that died in 2023 as a function of the total number of trees based on questionnaire data. (**d**) The map shows the absolute differences between the observed county-level dieback values and the expected values derived from the regression equation in panel (**c**). Darker shades indicate counties where the deviation from the national trend estimated from the questionnaire data is larger. (**e**) Mean annual temperature forecast for 2071–2100 (right) (Redrawn figure based on the HungaroMet Database (Hungarian Meteorological Service 2024; Lennert et al. 2024)

Based on validated answers, we received 888 responses from 329 settlements, representing over 10% of Hungary's settlements (3155 total). Responses were received from all counties, providing a reliable picture of spruce mortality in the Carpathian Basin (Fig. 3a). 6.0% of surveys were completed incorrectly. The high response rate reflects public sensitivity to accelerated climate events. As Gould et al. (2024) noted, extreme climate events motivate people to take action.

In 60 settlements (18.2%), no spruce deaths or damage were reported, while 269 settlements (81.8%) reported such cases. Respondents reported a total of 4081 (± 22) spruce trees before 2023, of which 2422 (± 20) died or showed signs of disease in 2023, equating to a 59.1% (± 1.5) mortality rate (Supplementary Material Table S1, Supplementary Material S5).

The relationship between the total number of reported trees and the number of dead trees is shown in Fig. 3c. Figure 3d maps the absolute deviations of county-level observations from this regression (Spruce tree data: Supplementary Material S5). Counties with larger deviations generally correspond to areas with higher dieback. The spatial patterns in Fig. 3b and d are consistent, indicating that high-mortality regions are not randomly distributed.

The dieback rates reported by respondents reveal the following significant pattern (*p* < 0.05). High mortality rates (60–77%) were reported in areas (counties) where the mean summer temperature was high between 1991 and 2020 (ρ = 0.46, τ = 0.34) and reached the 23 °C threshold in 2022 (ρ = 0.49, τ = 0.35) (cf. Figure 3b and Fig. 2b, c). The number of dead spruce trees showed a significant correlation between the summer temperature anomaly exceeding 1 °C in 2022 (ρ = 0.48, τ = 0.35) (cf. Figure 3b and Fig. 2d, e). Additionally, a significant negative correlation existed between the rate of dead spruce trees and the sum of the mean summer precipitation between 1991–2020 (ρ = -0.50, τ = -0.34), as well as the total annual precipitation in 2022 (ρ = -0.52, τ = -0.36) and in 2023 (ρ = -0.51, τ = -0.35) (cf. Figure 3b and Fig. 2F, g). Notably, the regions with the highest dieback rates coincide with those predicted to be most heat-sensitive based on the annual temperature forecast (cf. Figure 3d and e).

The mean temperature and precipitation during the growing seasons of the past decades (1991–2020) also relate to the level of spruce mortality, but the mean summer temperature and annual precipitation in 2022 can also be influential factors. The low precipitation in 2023 may further reduce the likelihood of spruce tree survival. Jevšenak et al. (2021) and Černý et al. (2020) examined extensive Slovenian and Czech spruce forests. Although clear differences exist between the responses of forest and urban individuals, their studies likewise identified summer temperature as a key factor influencing spruce.

The increased street and garden spruce mortality observed in the Carpathian Basin in 2023 represents an intensification of a long-term trend that has affected Hungarian planted spruce stands for decades (Supplementary Material S3 and S6). In the forest areas for forestry purposes, the area of conifer species (which roughly equal with spruce forests) decreased by half between 2000 and 2022 (Supplementary Material Fig. S1a). Norway spruce prefers macroclimatic conditions with a mean annual temperature of approximately 6 °C (Tjoelker et al. 2007), whereas the mean annual temperature in the Carpathian Basin has risen from around 10 °C to 11.5 °C. This warming trend is pushing the region’s climate beyond the tolerable threshold for Norway spruce, placing the species at the edge of its such a kind of range, where the species is not only non native but not viable anymore. Consequently, the decline of spruce serves as a sensitive indicator of accelerated climate change (Molnár and Végvári 2016).

The summer of 2023 was exceptionally hot across the Northern Hemisphere (Esper et al. 2024), weakening tree physiology and reducing natural resilience to pests throughout Europe, also in the native spruce forests (Kunert et al. 2022). For a European overview of Norway spruce decline, see Supplementary Material S4. The frequency, intensity, duration, and spatial extent of heatwaves are increasing in Europe, but the pattern of the phenomenon is uneven (Pardo and Paredes-Fortuny 2024). In general, there is no doubt that in basins (such as the more continental climate Carpathian Basin), the symptoms of climate change appear earlier and more intensively compared to non-basin regions; thus, their monitoring is particularly timely (Bianucci et al. 2023; Isinkaralar et al. 2024).

Although our dataset provides nationwide coverage, several possible limitations need to be considered. First, internet access and online engagement are likely to be lower in some regions, particularly in smaller rural settlements. Residents of these areas may therefore have been less likely to encounter the questionnaire. As the study did not aim to analyse such social or demographic aspects, we did not attempt to correct for these differences. Importantly, every county also contains towns and cities where online participation is substantially higher, which partly mitigates this potential bias.

Second, we assumed that the planting of Norway spruce as a garden or park tree is relatively uniform across the country. This assumption may not hold everywhere; however, expressing mortality as the proportion of reported dead trees helps to reduce the influence of regional differences in planting intensity. It is also possible that some dead trees were reported more than once. Detecting and removing duplicate submissions was challenging, and we did not restrict responses based on IP (Internet Protocol) address. Such restrictions would require processing personal data (IP addresses), which raises GDPR (General Data Protection Regulation) considerations and could also unintentionally block legitimate submissions from shared municipal networks.

Nevertheless, counties encompass substantial heterogeneity in altitude, urbanization, land-use patterns, and settlement structure. These factors can influence both local climate conditions and the likelihood or severity of spruce decline. As a result, county-level aggregation inevitably smooths out fine-scale variation, potentially obscuring relationships that operate at smaller spatial scales. Given the nationwide scope of the study and the availability of high-resolution gridded climate data (0.1° daily temperature and precipitation from odp.met.hu), we consider county-level averaging a reasonable compromise that captures broad climatic patterns while maintaining consistency between datasets.

In conclusion, the warming climate with extreme hot summers can lead to rapid losses of spruce trees in parks and gardens of the Carpathian Basin. The accelerated Norway spruce dieback is a clear sign of climate change unfolding before us. This highlights that climate-induced ecological changes can happen faster than previously thought. Spruce tree dieback in the Carpathian Basin indicates that continental basins are at heightened climate risk. Our results support the view that Norway spruce can illustrate the broader ecological and economic consequences of climate-driven species loss. Until global solutions to climate change are achieved, locally adapted actions, supported by regional, high-resolution climate information, will remain essential.

## Authorship contribution

Zsuzsa Lisztes-Szabó: Conceptualization, Formal analysis, Investigation, Methodology, Project administration, Resources, Visualization, Writing – original draft, Writing – review and editing. Albert Tóth: Data curation, Methodology, Writing – review and editing. Anna F. Filep: Investigation, Project administration, Writing – review and editing. Olivér Szentes: Data curation, Software. Elemér László: Data curation, Methodology, Software, Visualization, Writing – review and editing. Mihály Braun: Conceptualization, Data curation, Investigation, Methodology, Visualization, Writing – review and editing.

## Supplementary Information

Below is the link to the electronic supplementary material.

Supplementary file1 (DOCX 162 KB)

Supplementary file2 (XLSX 58 KB)

Supplementary file3 (XLSX 20 KB)

## Data Availability

The raw dataset analyzed in the current study is not publicly available because it is still under further analysis from a different perspective. However, it is available from the corresponding author upon reasonable request. The derived data generated during this study are included in this published article and its Supplementary information files.
