# Supplementary material for: Climate-driven decline of Norway spruce in Central Europe: a threshold crossing in the warming Carpathian Basin
Source: Int J Biometeorol. 2026 Apr 14;70(4):121. doi: 10.1007/s00484-026-03174-9 (PMC13079526; doi:10.1007/s00484-026-03174-9)
Supplement: Supplementary file 1 — Supplementary file1 (DOCX 162 KB) [file 484_2026_3174_MOESM1_ESM.docx]

Supplementary Material

International Journal of Biometeorology

**Climate-driven decline of Norway spruce in Central Europe: A threshold crossing in the warming Carpathian Basin**

Zsuzsa Lisztes-Szabó^a,b^, Albert Tóth^a^, Anna F. Filep^c^, Olivér Szentes^d^, Elemér László^a^, Mihály Braun^a^

^a^HUN-REN Institute for Nuclear Research, Bem tér 18/c, Debrecen, 4032-Hungary

^b^Department of Botany, Faculty of Science and Technology, University of Debrecen, Egyetem tér 1, Debrecen, 4032-Hungary

^c^Pál Juhász-Nagy Doctoral School of Biology and Environmental Sciences, University of Debrecen, Egyetem tér 1, Debrecen, 4032-Hungary

^d^Hungaromet Hungarian Meteorological Service, Climate Research Department, Kitaibel Pál u.1, Budapest 1024, Hungary

Corresponding author address: HUN-REN Institute for Nuclear Research, Bem tér 18/c, Debrecen, H-4032, Hungary

Corresponding author email address: lsz.zsuzsa@atomki.hu

**Supplementary Material S1.**

***Picea* (spruce) species are distributed in the northern hemisphere with several varieties and forms** (Schmidt-Vogt 1977)**.**

Some taxons have unclear taxonomical rank. Species with restricted and geographically isolated ranges are marked with an asterisk.

Many *Picea* species grow in the mountains at 1400–3600 m altitudes. They are also considerable species in the local forest plant communities and are economically important.

**Europe**

*P. abies* (L.) KARST.

*P. obovata* LEDEB.

**P. omorica* (PANČIĆ) PURKYNE

**North America**

P*. engelmannii* (PARRY) ENGELM.

*P. glauca* (MOENCH) VOSS

*P. mariana* (MILL.) BRITT.

*P. pungens* ENGELM.

*P. rubens* SARG.

*P. sitchensis* (BONG.) CARR.

**P. breweriana* S. WATS.

**P. mexicana* MARTINEZ

**P. chihuahuana* MARTINEZ

**Northern and northeastern Asia**

*P. jezoensis* (SIEB. et ZUCC.) Carr.

*P. obovata* LEDEB.

**P. alcoquiana* (VEITCH ex LINDL.) CARR.

**P. glehnii* (FR. SCHMIDT) MAST.

**P. koraiensis* NAKAI

**P. koyamai* SHIRASAWA

**P. maximowiczii* REGEL ex MAST.

**P. polita* (SIEB. et ZUCC.) CARR.

**Southeastern Asia**

**P. asperata* MAST.

**P. aurantiaca* MAST.

**P. brachytyla* (FRANCH.) PRITZEL

**P. crassifolia* KOMAROW

**P. farreri* PAGE et RUSHF.

**P. likiangensis* (FRANCH.) PRITZ.

**P. meyeri* REHD. et WILLS.

**P. morrisonicola* HAYATA

**P. neoveitchii* MAST.

**P. purpurea* MAST.

**P. retroflexa* MAST.

**P. wilsonii* MAST.

**Central Asia** (Himalayas)

**P. spinulosa* (GRIFF.) HENRY

**P. schrenkiana* FISCH et C.A. MEY.

**P. smithiana* (WALL.) BOISS.

**Caucasus**

**P. orientalis* (L.) LINK

**References**

Tjoelker MG, Boratyński A, Bugała W. (ed.) 2007. Biology and Ecology of Norway spruce. Dordrecht, Springer.

Schmidt-Vogt H. 1977. Die Fichte. Hamburg, Verlag Paul Parey.

**Supplementary Material S2.**

**Material and methods**

**Weather extremity in 2022 and 2023 in the studied region (Carpathian Basin - Hungary)**

In this study, we focused on the Carpathian Basin, where in 2023, the sudden accelerated destruction of spruce trees became noticeable in the streets, parks, and gardens. We were wondering how the 2023 rapid Norway spruce dieback was quantifiable for the area and how it assoviates to the severity of the hot and drought. In Central Europe, and especially in the Carpathian Basin with a more continental climate, Norway spruce trees have been exposed to drought stress in previous years (and actually in decades) because of the hot summers and dry seasons (Kunert et al. 2022). According to Köppen’s classification, the Carpathian Basin (Hungary) belongs to the climate type of the warm-temperate climate zone with an even distribution of precipitation and hot summers (Geiger 1961). In the following, we rely on weather data from HungaroMet (2024) for Hungary. The mean temperature in 2023 was 12.2°C. This exceeded the 1991–2020 climate norms by 1.5 degrees on a national average. The summer in 2023 was 0.8 °C warmer than the long-term national average (1991–2020). Summer maximum temperature was often run close to 40 °C, atmospheric conditions were with low humidity, simultaneously with increased UV radiation, and the soil was water-poor. The national average of the annual mean temperature rises significantly at the 90% confidence level based on the linear trend estimation of the long-time series starting from 1901 (from the official start of recording temperature data in the country). Its change over the last 123 years (between 1901 and 2023) was an average of +1.5 °C, while within the country, there was a temperature change between at least +1.2 °C and at most +1.9 °C (HungaroMet, 2024).

**Methods**

Accurate counting was hindered by the fact that dead trees in streets, parks, and gardens are usually processed and removed without delay. To be able to estimate it soon and directly, we collected the contact information of the local municipal governments of Hungarian settlements and towns, asking them to disseminate a Google questionnaire, which we asked the citizens to fill out. The Hungarian Central Statistical Office (https://www.ksh.hu) assisted us, via its “Contact us” system (case ID: 20240111-120206), in obtaining the information necessary to contact local municipalities. They directed us to the source containing the e-mail addresses of mayoral offices and notaries for all settlements. These data were available through the National Statistical Data Collection Programme (#1 http). By selecting the item entitled “OSAP 1621 Municipal Master Data Collection – Data Tables for 2022”, the relevant files could be downloaded in compressed format (direct link provided in the References). Using these data, we sent a total of 6,394 outreach e-mails to local municipalities—specifically to the mayoral offices and the notaries (Table S1). Thus, each settlement received two e-mails, one addressed to each of these administrative units. A total of 69 e-mail addresses proved to be invalid or resulted in bounced messages. However, we did not identify any municipality for which both contact addresses failed; in every case, at least one of the two e-mails reached its destination. Where possible, municipalities shared the questionnaire through their communication channels. In the case of small settlements, the questionnaire was typically completed directly by the municipal offices using information about their own settlement. The questions related to how many spruce trees lived around the interviewee’s household (in the garden or the street), and how many died or showed signs of destruction during 2023. The local governments of towns and settlements included spruce losses in cemeteries and parks in the questionnaire. In addition, we collected the postal code of the interviewee’s place of residence so that we can assign data to counties for county-level comparison. The data collected by the questionnaires therefore refer to trees in gardens, on streets, in cemeteries, and in parks. One month was available to fill out the form (January 2024). The incorrectly completed forms were filtered out. The following types of errors occurred in the submitted responses: some respondents provided text answers without any numerical data; in other cases, the number of trees was reported as an area unit (likely referring to forested land); or the reported number of dead trees exceeded the total number of trees. For the statistical evaluation, we chose the answers where the number of dead trees was larger than or equal to 1. We also excluded data for some records reporting more than 50 specimens, as we interpreted these as potential forest monocultures. In such cases, we verified the land cover on Google Maps to confirm whether the site was indeed a monoculture. Trees growing in spruce stands may respond differently to climatic factors, and spruce plantations in the Carpathian Basin were historically established in specific climatic settings. For these reasons, including such stands would have introduced multiple sources of bias into our results. Uncertainties in the total and dead number of trees were estimated using the square root of the sum of the reported values. Subsequently, error propagation was applied to determine the uncertainty in the ratio of dead trees (Table S1).

We examined the correlation between the rate of spruce death (number and percentage of dead trees) and meteorological variables: mean annual and summer temperature, precipitation between 1991 and 2020, and in 2022 and 2023, by the county. Temperature and precipitation anomalies were calculated based on differences from the 1991–2020 period. Spearman's and Kendall's rank correlation tests were applied (Past Version 2.17c, Hammer et al. 2001). The data and the test results are in Supplementary Material S5.

Additionally, as part of the broader analysis, statistical data on planted Norway spruce stands in Hungary were reviewed and discussed. These data and their interpretation are included in Supplementary Materials S3 and S6.

**References**

Geiger R. 1961. Überarbeitete Neuausgabe von Geiger, R.: KöppenGeiger / Klima der Erde. (Wandkarte 1:16 Mill.). Gotha, KlettPerthes.

Hammer O, Harper DAT, Ryan PD 2001. PAST: paleontological statistics software package for education and data analysis. Palaeontologia Electronica 4(1): 9.

Hungarian Meteorological Service 2024. HungaroMet Database. Mean summer temperatures, temperature anomaly and standardized precipitation index in Hungary in 2022 and in 2023. Yearly mean temperature forecast for 2071–2100. <https://www.met.hu/eghajlat/magyarorszag_eghajlata/eghajlati_visszatekinto/elmult_evszakok_idojarasa/> Viewed 22. 06. 2025.

Kunert N, Hajek P, Hietz P, Morris H, Rosner S, Tholen D (2022) Summer temperatures reach the thermal tolerance threshold of photosynthetic decline in temperate conifers. Plant Biol 24:1254–1261. https://doi.org/10.1111/plb.13349

OSAP 1621 Municipal Master Data Collection – Data Tables for 2022’,. <http://kormanyhivatal.hu/download/f/08/88000/OSAP1621_2022.zip>.

#1 http://kormany.hu/miniszterelnokseg/orszagos-statisztikai-adatfelveteli-program

**Table S1.** Summary of valid answers. Total number of outreach e-mails (@), number of answers (n), total number of reported *P. abies* (N total), number of trees dead in 2023 (N dead), percentage of dead spruce trees (Dead %).

| **County**  **(Hungarian)** | **@** | **n** | **N total** | **±Unc** | **N dead** | **±Unc** | **Dead (%)** | **±Unc** |
| --- | --- | --- | --- | --- | --- | --- | --- | --- |
| Bács-Kiskun | 240 | 19 | 146 | 12 | 51 | 7 | 34.9 | 5.7 |
| Baranya | 604 | 25 | 229 | 15 | 107 | 10 | 46.7 | 5.5 |
| Békés | 152 | 103 | 498 | 22 | 386 | 20 | 77.5 | 5.3 |
| Borsod-Abaúj-Zemplén | 718 | 26 | 304 | 17 | 153 | 12 | 50.3 | 5.0 |
| Budapest (Capital) | 48 | 17 | 87 | 9 | 43 | 7 | 49.4 | 9.2 |
| Csongrád | 122 | 33 | 180 | 13 | 108 | 10 | 60.0 | 7.3 |
| Fejér | 218 | 38 | 166 | 13 | 128 | 11 | 77.1 | 9.1 |
| Győr-Moson-Sopron | 368 | 5 | 75 | 9 | 52 | 7 | 69.3 | 12.5 |
| Hajdú-Bihar | 166 | 21 | 141 | 12 | 85 | 9 | 60.3 | 8.3 |
| Heves | 244 | 19 | 194 | 14 | 117 | 11 | 60.3 | 7.1 |
| Jász-Nagykun-Szolnok | 158 | 64 | 325 | 18 | 231 | 15 | 71.1 | 6.1 |
| Komárom-Esztergom | 154 | 16 | 105 | 10 | 57 | 8 | 54.3 | 8.9 |
| Nógrád | 264 | 4 | 48 | 7 | 20 | 4 | 41.7 | 11.1 |
| Pest | 376 | 81 | 444 | 21 | 276 | 17 | 62.2 | 4.8 |
| Somogy | 494 | 11 | 122 | 11 | 39 | 6 | 32.0 | 5.9 |
| Szabolcs-Szatmár-Bereg | 460 | 36 | 216 | 15 | 114 | 11 | 52.8 | 6.1 |
| Tolna | 220 | 10 | 78 | 9 | 52 | 7 | 66.7 | 11.9 |
| Vas | 434 | 35 | 436 | 21 | 252 | 16 | 57.8 | 4.6 |
| Veszprém | 436 | 15 | 160 | 13 | 93 | 10 | 58.1 | 7.6 |
| Zala | 518 | 7 | 127 | 11 | 58 | 8 | 45.7 | 7.2 |
| **Total** | **6394** | **585** | **4081** | **64** | **2422** | **49** | **59.3** | **1.5** |

**Supplementary Material S3****: A perspective on planted spruce forests in Hungary**

Increased mortality of planted spruce in 2023 is the sharp intensification of a previous trend that has affected planted forests for decades. In the center of the Carpathian Basin, in Hungary, the areal status of forests is as follows according to the most recent and available data of the Hungarian Central Statistical Office (https://www.ksh.hu). In 2022, the forest area covered with coniferous trees was 175,800 hectares, and the majority of spruce trees were planted in the period after 1960. Spruce covered 13,100 hectares in 2019. In the forest areas for forestry purposes, the area of conifer species (Scots and black pine [*Pinus sylvestris*, *P.* *nigra*] plantations excluded) decreased by half between 2000 and 2022 (from 28,500 hectares, Fig. S1a). Due to dried or diseased spruce stands, the annual logging of all the conifer species stands increased from 866 thousand cubic meters to 1,066 thousand cubic meters between 1996 and 2022 (Fig. S1b). The relevant data are in Supplementary Material S6: Table S2, Table S3, and Table S4. The data demonstrate that Hungary is already undergoing a major spruce dieback.


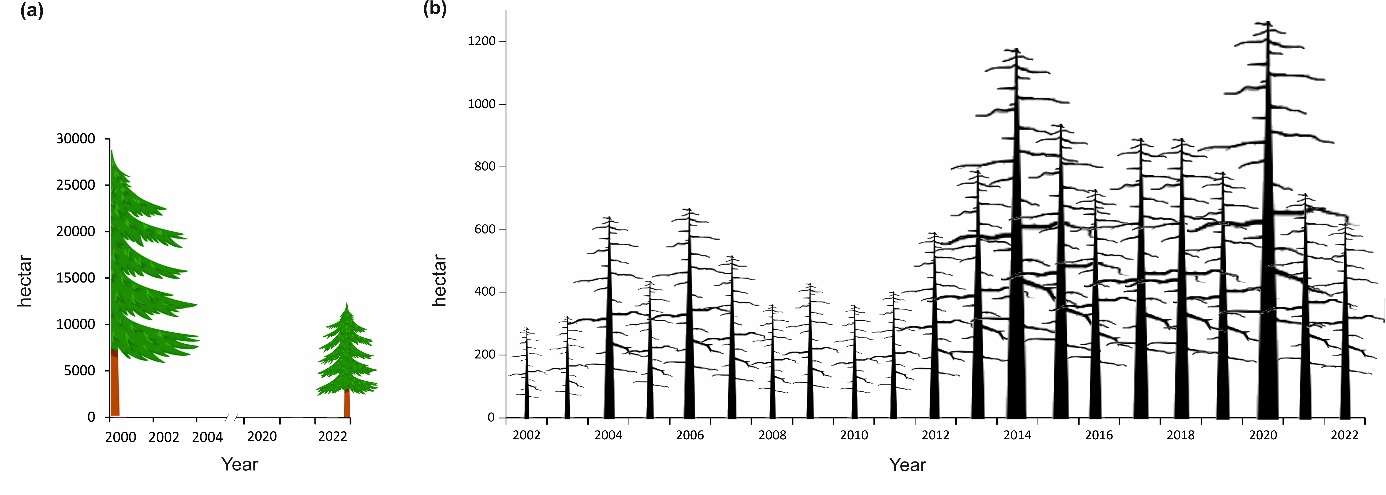


**Figure S1.** Decreased forest areas for forestry purposes in the Carpathian Basin (Hungary). (a) The area of conifer stands in the forest areas (Scots and black pine [*Pinus sylvestris*, *P. nigra*] plantations excluded) between 2000 and 2022. (The intensity of spruce decline for 2023 is estimated by the survey, Fig. 4a) (b) Annual increase in the area of conifer stands logged due to drought or disease between 1996 and 2022. (For detailed data, see Supplementary Material S6, Table S2, Table S3, and Table S6.) The data source was the available data of the Hungarian Central Statistical Office (https://www.ksh.hu).

**References**

Hungarian Central Statistical Office; https://www.ksh.hu. (Referenced forest data source.) Viewed 22. 06. 2025.

**Supplementary Material S4**

**The European overview of Norway spruce decline**

However, the summer of 2023 was dramatically hot not only in Central Europe. Esper et al. (2024) analyzed the June-August surface air temperatures combining the observed and reconstructed data to show that 2023 was the warmest Northern Hemisphere extra-tropical summer over the past 2000 years, exceeding the 95% confidence range of natural climate variability by more than half a degree Celsius.

Climatic adversities, especially extremely hot and/or drought circumstances, induce a weakened physiological condition of the trees, hence reducing natural resilience against pests and insects (Kunert et al. 2022). Norway spruce roots are shallow, drawing moisture from the upper 50 cm of soil, making them vulnerable to drought. Additionally, warmer temperatures and expanded mild zones in high mountains promote the spread of pests, leading to pest gradations (Tjoelker et al. 2007). Infected trees in built environments can serve as bridges for parasites to spread to larger spruce stands. Weakened trees may dry out or be uprooted by storms, posing a risk of accidents in urban environments.

Increased spruce mortality is a characteristic of the entire Europe (Jääskeläinen et al. 2025; Jandl 2020; Vancura 1995; Wolf 1995). Simultaneously, the possibilities for the recreation of young spruce stands are reduced, and the reforestation intensity of this species decreased, especially outside its natural range (OECD 2006). In the past, significant tree damage occurred only after storms and natural disasters, especially in groves planted at a lower height. In recent years, however, it has become increasingly common in higher regions and native areas. In the 1990s, a bark beetle infection caused the decay of spruce forests in the central part of the Sumava Mountains, the Czech Republic, bordering the Bavarian Forest National Park, Germany, where the bark beetle infection started in the late 1980s (Hais and Kučera 2008). Jandl (2020) reported the destruction of spruce forest stands in Austria, and the weakened spruce trees destroyed by bark beetles or storms, also in Germany and Slovakia. In Romania, Pócs (2022) already observed the destruction of spruce trees in 2009 (Bihor Mountains, Romania), in the optimum spruce zone, at an altitude of 1300–1400 m.

Finally, if we look even wider, it is important to note that the *Picea* genus has species living in the sensitive zone near the forest and tree line, with an endangered conservation status. Serbian spruce (*Picea omorika*) is an endangered and dragon spruce (*Picea asperata*) is a vulnerable IUCN Red List species (Carter and Farjon 2013; Aleksić et al. 2017). However, we can find 18 further *Picea* species on the List with marks of decreasing individual counts.

**References**

Aleksić JM, Ballian D, Isajev D, et al. 2017. *Picea omorika*. The IUCN Red List of Threatened species 2017: e.T30313A84039544. <https://dx.doi.org/10.2305/IUCN.UK.2017-2.RLTS.T30313A84039544.en>. Viewed 18 July 2024.

Carter G and Farjon A. 2013. *Picea asperata*. The IUCN Red List of Threatened Species 2013: e.T42320A2972242. https://dx.doi.org/10.2305/IUCN.UK.2013-1.RLTS.T42320A2972242.en. Viewed 18 July 2024.

Jandl R. 2020. Climate-induced challenges of Norway spruce in Northern Austria. Trees For People 1: 100008.

Jääskeläinen J, Junttila S, O’Sullivan H, Cheng Y, Horion S, Vastaranta M. 2025. Quantifying the drivers of tree mortality: A case study from urban recreational boreal forest. Urban Forestry & Urban Greening, 104 (2025) 128672.

Kunert N, Hajek P, Hietz P, Morris H, Rosner S, Tholen D (2022) Summer temperatures reach the thermal tolerance threshold of photosynthetic decline in temperate conifers. Plant Biol 24:1254–1261. https://doi.org/10.1111/plb.13349

Vancura K. 1995. Conservation of Norway spruce genetic resources in the Czech Republic. In: Turok et al. (Eds.), *Picea abies* Network. Report of the first meeting, 16-18 March 1995, Tatra National Park, Stara Lesna, Slovakia. Rome, Italy, International Plant Genetic Resources Institute.

Wolf H. 1995. The conservation of Norway spruce genetic resources in the Federal Republic of Germany. In: Turok et al. (Eds), *Picea abies* Network. - Report of the first meeting, 16-18 March 1995, Tatra National Park, Stara Lesna, Slovakia, Rome, Italy, International Plant Genetic Resources Institute.
